# Ultrafast Polymerization of a Self-Adhesive and Strain Sensitive Hydrogel-Based Flexible Sensor for Human Motion Monitoring and Handwriting Recognition

**DOI:** 10.3390/polym16111595

**Published:** 2024-06-04

**Authors:** Bin Du, Mengwei Yin, Kenan Yang, Sainan Wang, Yiting Pei, Rubai Luo, Shisheng Zhou, Huailin Li

**Affiliations:** 1 Faculty of Printing, Packaging Engineering and Digital Media Technology, Xi’an University of Technology, Xi’an 710054, China; dubin@xaut.edu.cn (B.D.); 19862128711@163.com (M.Y.); m18110737432@163.com (S.W.); p13992388332@163.com (Y.P.); zhoushisheng@xaut.edu.cn (S.Z.); lihuailin@xaut.edu.cn (H.L.); 2 Shaanxi Provincial Key Laboratory of Printing and Packaging Engineering, Xi’an University of Technology, Xi’an 710054, China; 1200210002@stu.xaut.edu.cn; 3 School of Mechanical and Precision Instrument Engineering, Xi’an University of Technology, Xi’an 710054, China

**Keywords:** hydrogel, adhesion, flexible sensor, wearable electronic device

## Abstract

Hydrogel-based flexible electronic devices have great potential in human motion monitoring, electronic skins, and human-computer interaction applications; hence, the efficient preparation of highly sensitive hydrogel-based flexible sensors is important. In the present work, the ultrafast polymerization of a hydrogel (1–3 min) was achieved by constructing a tannic acid (TA)-Fe^3+^ dynamic redox system, which endowed the hydrogel with good adhesion performance (the adhesion strength in wood was 17.646 kPa). In addition, the uniform dispersal ensured by incorporating polydopamine-decorated polypyrrole (PPy@PDA) into the hydrogel matrix significantly improved the hydrogel’s stretching ability (575.082%). The as-prepared PAM/CS/PPy@PDA/TA hydrogel-based flexible sensor had a high-fidelity low detection limit (strain = 1%), high sensitivity at small strains (GF = 5.311 at strain = 0–8%), and fast response time (0.33 s) and recovery time (0.25 s), and it was reliably applied to accurate human motion monitoring and handwriting recognition. The PAM/CS/PPy@PDA/TA hydrogel opens new horizons for wearable electronic devices, electronic skins, and human-computer interaction applications.

## 1. Introduction

Flexible and wearable electronic devices have great potential in health monitoring, electronic skins, and human-computer interaction applications due to their flexibility, portability, and comfort [1,2,3,4,5,6]. Although conventional inorganic conductive materials, such as metals, metal oxides, and nanomaterials, have excellent electrical conductivity, their limited flexibility results in poor contact between electronic devices and the tissue surface [7]. Hydrogels are three-dimensional hydrophilic polymer cross-linking materials with remarkable stretchability and excellent adhesion and self-healing properties [8,9,10,11]. Conductive hydrogels combine the excellent properties of conductive fillers (such as conductive polymers [12,13,14], metal particles [15,16], and carbon nanomaterials [17,18,19]) and hydrogels; thus, they are promising candidates for flexible electronic devices, such as soft robots, electronic skins, and implantable devices [20,21,22]. Due to the excellent electrical conductivity of metal compounds, hydrogels offer excellent conductivity by embedding the metals or metal NPs in the hydrogel structure [23]. Carbon nanomaterials have good electrical conductivity and the ability to improve the mechanical properties of conductive hydrogels [24]. However, these conductive materials are prone to leaking from the hydrogel matrix due to insufficient sealing, which limits their practical application in conducting hydrogels [12]. Conjugated π conductive polymers have high crosslinking ability, which hinders their leakage from the network [12]. In addition, they have high conductivity, biocompatibility, and mechanical flexibility. Wang et al. [25] have proposed to organize poly(3,4-ethylenedioxythiophene)-poly(styrene sulfonate)(PEDOT:PSS) into a nanoscale network using conductive cellulose nanofiber (CNF) as a template and fabricating ultra-stretchable and highly sensitive conductive hydrogel. The CNF/PEDOT:PSS/PAM hydrogel exhibited an ultimate tensile strain of 1881% and toughness of 3.72 MJ/m^3^, The sensitivities of the hydrogel (GF) in strain ranges of 0–10%, 10–300%, and 300–1100% were 1.35, 1.76, and 5.16, respectively. To accurately monitor high-intensity motion changes and weak vibration signals, this requires that hydrogels need to have high sensitivity under small strains. Researchers are constantly investigating and improving the types of conductive fillers used in conductive hydrogels and their conductive mechanisms [26,27].

Conductive polymers utilize conjugated structures to provide electron transport, making it easier to convert them into electronic signals, which enforces the high sensitivity of electronic hydrogels under fine strains [21]. Polypyrrole (PPy), as a typical conductive polymer, is extensively used in conductive hydrogel-based sensors because it is lightweight, requires simple synthesis conditions, and has a wide conductivity variation range [28,29,30,31]. However, due to the inherent conjugated heterocyclic structure of PPy, it cannot be dispersed uniformly in aqueous solutions, preventing the complete bonding of PPy with hydrophilic hydrogels [32]. Polydopamine (PDA) is a substance with a structure similar to the adhesion protein of mussels [33], and catechol groups in its molecules can form complexes with conductive polymers through different types of interactions. In addition, due to the existence of carboxyl, imine, amino, and phenol groups, PDA has high hydrophilicity [34,35]. Thus, PDA is often used as a surface modification material. Duo et al. [36] synthesized MSNs-DOX@PDA-PEG composite nanocarriers by directly encapsulating DOX-loaded MSN with PDA and polyethylene glycol (PEG). Deng et al. [37] used PDA to modify CNTs and doped them into acrylamide-co-acrylic acid polymers to prepare conductive hydrogels with good adhesion, and it was noticed that the incorporation of PDA allowed CNTs to be uniformly dispersed in the hydrogel system, resulting in high electrical conductivity. Hence, PDA is expected to be a hydrophilic carrier of PPy.

The preparation of conductive hydrogel-based wearable and flexible electronic devices is time-consuming [38,39], and they often need to be attached to the tissue surface using tapes or adhesives. However, due to this unstable contact, wearable electronic devices cannot accurately identify signals. Conductive hydrogels with self-adhesive properties can tightly adhere to the tissue surface, and a stable interface can be established between wearable electronic devices and the tissue surface, realizing precise signal monitoring [40,41]. Therefore, problems of the efficient preparation of conductive hydrogel-based sensors with good adhesion properties still need to be solved. In recent years, inspired by natural mussel adhesion, self-adhesive sensors based on catechol chemical binders have been proposed [42,43,44,45]. Tannic acid (TA) is a low-cost and biocompatible plant-derived polyphenol, and it has a catechol-like moiety with 25 phenolic hydroxyl groups in a single molecule, forming non-covalent hydrogen bonding sites with other substances in the system [46]. In addition, due to the reductive and electronegative characteristics of the polyphenol structure, TA can undergo metal coordination with metal ions, leading to a significant increase in the stability of substances. Wilker et al. [47] reported that the tri-dopa-Fe^3+^ complex underwent metal coordination through a reduction of Fe^3+^ to Fe^2+^ and the phenolic hydroxyl groups in dopa were oxidized to semiquinone radicals. Based on mimetic mussel chemistry, Jia et al. [48] proposed a dual autocatalytic system consisting of transition metal ions and catechol-based molecules. This system was effective in initiating free radical polymerization without any external stimuli and became gelatinized within 5 s at 6 °C. The synergistic interaction between polyphenols and transition metal ions opens a new path for the efficient preparation of adhesive hydrogels.

In the present research, a highly efficient fabrication method of stretchable, self-adhesive, and strain-sensitive hydrogels was proposed. A composite hydrogel was prepared by in situ polymerization using PPy@PDA, chitosan (CS), TA, and acrylamide (AM). The ultrafast polymerization of the hydrogel (1–3 min) was realized at room temperature. The hydrogel had satisfactory mechanical properties with good adhesion to glass, wood, filter papers, rubber, and human skin. The flexible and wearable sensor assembled from the hydrogel had a low detection limit (strain = 1%), high sensitivity at small strains (GF = 5.311 at strains = 0–8%), and fast response time (0.33 s) and recovery time (0.25 s). The as-fabricated sensor was reliably applied to accurately monitor human movements and handwriting recognition. When the sensor was assembled into a stylus, it successfully manipulated a cell phone screen. These results indicate that the as-prepared hydrogel has great prospects in wearable electronic devices, electronic skins, and human-computer interaction applications.

## 2. Materials and Methods

### 2.1. Materials

Acetic acid was purchased from Tianjin Fuyu Fine Chemical Co., Ltd. (Tianjin, China). Sodium dodecylbenzene sulfonate (SDBS) and FeCl_3_·6H_2_O were procured from Tianjin Damao Chemical Reagent Factory. (Tianjin, China). Tannic acid was obtained from Tianjin Tianli Chemical Reagent Co., Ltd. (Tianjin, China). Tris-Hydrochloride buffer solution was purchased from Shanghai Aladdin Biochemical Technology Co., Ltd. (Shanghai, China). Pyrrole (Py), dopamine hydrochloride (DA), Chitosan (CS, degree of deacetylation ≥95%, viscosity: ca. 100–200 mpa.s), Acrylamide (AM, AR, 99.0%), ammonium persulfate (APS), and N,N’-methylenebis(acrylamide) (MBA) were purchased from Shanghai McLean Biochemical Technology Co., Ltd. (Shanghai, China). All reagents were of analytical grade.

### 2.2. Preparation of the PPy@PDA Complex

First, 100 mL, of 2% acetic acid solution was mixed with 50 mL (0.003 mol) of SDBS and stirred in an ice-water bath. Subsequently, 700 μL of Py solution was added to the solution and stirred for 20 min. Then, 8.11 g FeCl_3_·6H_2_O was dissolved in 50 mL deionized water and added slowly to the resultant solution dropwise (15 drops/min), and reacted for 12 h at 0–8 °C. After the reaction, centrifugal washing was conducted to remove impurities, and finally, the product was dried in a vacuum drying oven at 55 °C for 12 h to obtain PPy.

A total of 0.5 g of PPy was added to 50 mL of Tris buffer (pH = 8.5) and ultrasonically dispersed for 30 min. Subsequently, DA (DA to PPy mass ratio = 1:2) was added to PPy, and polymerization was conducted at 30 °C for 24 h. Further repeated centrifugal washing to neutrality (12,000 rpm for 20 min) was performed to remove impurities, and the product was dried in a vacuum drying oven at 55 °C for 12 h to obtain the PPy@PDA complex powder.

### 2.3. Preparation of Hydrogels

First, 0.5 g of CS was dissolved in 100 mL, of 2% acetic acid solution to obtain CS solution. Subsequently, different amounts of the PPy@PDA complex (PPy@PDA/AM (wt%) = 0.3 wt%, 0.6 wt%, 0.9 wt%, and 1.2 wt%) were added to 20 mL of deionized water and sonicated for 10 min. Further, 60 mg of TA was added to the solution and sonicated for another 10 min to obtain the PPy@PDA/TA solution. The CS solution and the PPy@PDA/TA solution of the same mass were mixed well to obtain solution A. Further, 6 g of AM was dissolved in 12 g of deionized water, and then 60 mg of APS and 12 mg of MBA were added to the solution and stirred to obtain solution B. Finally, 15 mL of solutions A and B were mixed evenly, and 225 μL of 0.2 mol/L FeCl_3_·6H_2_O solution was added to the resultant solution and stirred for 30 s. The solution was then transferred to a mold and polymerized at room temperature to obtain the PAM/CS/PPy@PDA/TA hydrogel.

### 2.4. Characterization

#### 2.4.1. Hydrophilicity of Pure PPy and the PPy@PDA Complex

Two hundred milligrams of the PPy@PDA complex and pure PPy were dried in a vacuum drying oven at 55 °C, and then pressed into sheets by a tablet press. The hydrophilicity of pure PPy and the PPy@PDA complex was determined by measuring their water contact angles (WCAs) using a JY-PH6 contact angle measuring instrument.

#### 2.4.2. Spectroscopic Analysis

Fourier transform infrared (FTIR) spectrometry (IR Spiri, Shimadzu, Japan) was conducted to detect the infrared spectra of the samples in a wavelength range of 4000–500 cm^−1^. X-ray photoelectron spectroscopy (XPS) was used to analyze the chemical compositions of pure PPy and the PPy@PDA complex.

#### 2.4.3. Microstructural Analysis

Scanning electron microscopy (SEM; Hitachi SU-8010, Tokyo, Japan) was employed to characterize the morphology of the hydrogels.

#### 2.4.4. Mechanical Test

The mechanical properties of the hydrogels were examined at room temperature using an Instron 5500 universal tester, and at least five tests were conducted for each sample. Rectangular-shaped tensile samples (60 mm × 20 mm × 2 mm) were tested at a tensile speed of 80 mm/min. The tensile cycling test was performed at a constant loading rate of 80 mm/min and a recovery rate of 50 mm/min. In the compression test, cylindrical samples with a diameter of 30 mm and a height of 20 mm were used. The compressive cycling test was carried out at a constant loading rate of 10 mm/min and a recovery rate of 10 mm/min. Compression cycles were performed in a continuous manner with no interval between two cycles. The elastic moduli of the hydrogels were calculated from the slopes of their stress-strain curves in the linear range, where their toughness was obtained from the integral over the area of the tensile curves.

#### 2.4.5. Adhesion Test

The hydrogels were adhered to different substrate surfaces with a bonding area of 20 mm × 20 mm. The adhesive strengths of the hydrogels were calculated from the maximum load divided by the area of the adhesive overlap.

#### 2.4.6. Sensitivity Test

The sensing properties of the hydrogels in the tensile mode were measured by coupling an Instron 5500 universal tester with a Keithley 2461 graphical digital source meter. In order to quantitatively measure the resistance sensitivity of the PAM/CS/PPy@PDA/TA-0.9 wt% hydrogel to different strains, the hydrogel was encapsulated in two pieces of 2 cm wide VHB tapes, and a copper wire was drawn at both ends to form a simple strain sensor [49]. Then, clamped the sensor between two fixtures of an Instron 5500 universal tester for reciprocating cyclic stretching with a stretching rate of 60 mm/min and a clamping distance of 20 cm. The copper wires at both ends of the strain sensor were connected to the Keithley 2461 graphical digital source meter. The change in resistance of the hydrogels under strains was recorded in real time by the graphical digital source meter. The PAM/CS/PPy@PDA/TA-0.9 wt% hydrogel was selected as the sensor to monitor human movements and handwriting recognition, and its relative change in resistance was calculated as follows:

$$\Delta R/R_{0}(\%)=(R-R_{0})/R_{0}\times 100\%,$$

where $R_{0}$ is the initial resistance, and R is the real-time resistance.

## 3. Results and Discussion

### 3.1. Design Strategy and Characterization of the Hydrogel

**Figure 1:**
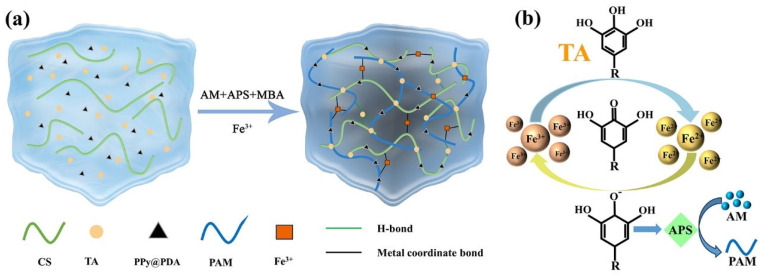
(**a**) Schematic diagram of the hydrogel preparation process and (**b**) tannic acid (TA)-Fe^3+^ dynamic redox system.

As conjugated polymers exhibit higher conductivity after doping, we used SDBS to dope PPy. Non-covalent interactions between PPy and PDA chains (π-π stacking and hydrogen bonding) [50] were used to modify the PPy surface by PDA, improving the dispersion of PPy in water. The well-dispersed PPy@PDA complex in the hydrogel matrix formed a homogeneous conductive gel network. A precursor solution was prepared by co-mixing the PPy@PDA complex, TA, and CS, and then introduced into the PAM gel substrate (Figure 1a). Finally, the dropwise addition of FeCl_3_·6H_2_O solution allowed hydrogen bonds and metal-ligand bonds in the polymer network to form a dynamic cross-linked network of CS, PAM, the PPy@PDA complex, and the TA-Fe^3+^ dynamic redox system. TA acted as a stabilizer and reducing agent and was rich in pyrogallol and catechol groups. The catechol groups in TA were oxidized to quinones, which were further converted to semiquinone radicals by oxidizing Fe^2+^ to Fe^3+^. The TA-Fe^3+^ dynamic redox system activated APS (the initiator) to generate free radicals at an increased rate, enabling the rapid formation of the PAM/CS/PPy@PDA/TA hydrogel at room temperature [51].

As PPy is hydrophobic, it does not integrate well with the hydrophilic polymer network in hydrogels [52], whereas PDA has a high affinity for both hydrophobic PPy and hydrophilic monomers [53]. Therefore, in the present work, PPy was modified with highly reactive and hydrophilic PDA to produce the PPy@PDA complex. The introduction of Fe^3+^ created a TA-Fe^3+^ dynamic redox system, which enabled the ultrafast polymerization of the hydrogel at room temperature.

### 3.2. Characterization and Hydrophilicity Analysis of the PPy@PDA Complex

**Figure 2:**
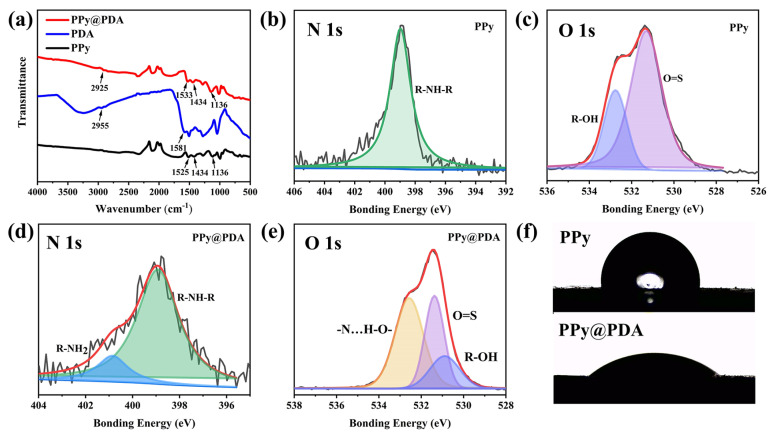
(**a**) FTIR spectrum of the PPy@PDA complex, (**b**,**c**) N 1s and O 1s photoelectron spectra of PPy, (**d**,**e**) N 1s and O 1s photoelectron spectra of the PPy@PDA complex, and (**f**) WCAs of PPy and the PPy@PDA complex.

The chemical structures of PPy, PDA, and the PPy@PDA complex were characterized by FTIR spectroscopy (Figure 2a). In the FTIR spectrum of PPy, the peaks at 1525 cm^−1^, 1434 cm^−1^, and 1136 cm^−1^ corresponded to the antisymmetric stretching vibration, symmetric stretching vibration, and C-N stretching vibration of the Py ring, respectively. The characteristic peaks of PDA appeared at 2955 cm^−1^ and 1581 cm^−1^ due to the C-H stretching and C=C stretching of aromatic side chains, respectively [54]. The PPy@PDA complex exhibited the characteristic peaks of both PPy and PDA, indicating the successful synthesis of the PPy@PDA complex. To further analyze the structure of the PPy@PDA complex, high-resolution XPS was adopted (Figure 2b–e) and the contents of each element were listed in Table 1. The nitrogen peak in the N 1s region of PPy was centered at 399 eV (Figure 2b), and it was attributed to the presence of nitrogen molecules in the Py ring (R-N-R). The N 1s peaks of the PPy@PDA complex had two components with N 1s split bond energies of 399 eV and 401 eV, (Figure 2d) due to the presence of (R-N-R) in the Py ring and R-NH_2_ in PDA, respectively. The O 1s photoelectron spectrum of PPy had two components: one centered at 531.3 eV and another at 532.8 eV (Figure 2c), corresponding to O=S bonds in SDBS and (R-OH) hydroxyl groups. The O 1s peaks of the PPy@PDA complex appeared at 530.9 eV, which corresponded to the (R-OH) hydroxyl groups, and at 531.4 eV and 532.6 eV, which corresponded to the O=S bonds in SDBS and -N⋯H-O- bonds, respectively (Figure 2e). The -N⋯H-O- bonds might be formed due to interactions between the N molecule of the Py ring and PDA [54,55,56]. In addition, the WCAs of PPy and the PPy@PDA complex were 97.2° and 39.1°, respectively (Figure 2f), indicating that the PPy@PDA complex was more hydrophilic than PPy.

### 3.3. Microstructure and Spectral Analysis of the PAM/CS/PPy@PDA/TA Hydrogel

**Figure 3:**
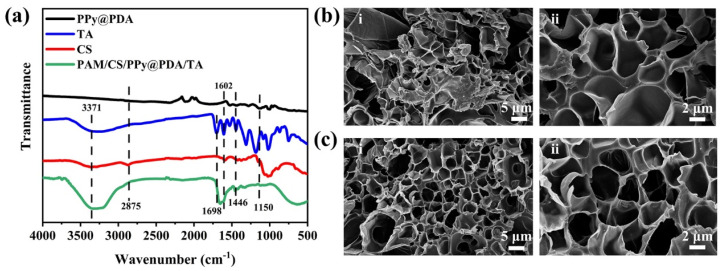
(**a**) FTIR spectra of the PAM/CS/PPy@PDA/TA hydrogel and (**b**,**c**) SEM images with 5 μm (i) and 2 μm (ii) magnification of the PAM/CS/TA hydrogel and the PAM/CS/PPy@PDA/TA hydrogel, respectively.

The FTIR spectra of TA, CS, the PPy@PDA complex, and the PAM/CS/PPy@PDA/TA hydrogel are shown in Figure 3a. In the FTIR spectrum of CS, the absorption peaks at 3371 cm^−1^, 2875 cm^−1^, and 1150 cm^−1^ corresponded to the O-H and N-H stretching vibrations, C-H stretching, and C-O-C bonding, respectively [57]. The typical characteristic absorption peaks of TA were detected at 3300 cm^−1^ (O-H), 1698 cm^−1^ (C=O), 1602 cm^−1^ (in-ring aromatic), and 1446 cm^−1^ (in-ring aromatic) [58]. In the FTIR spectrum of the PAM/CS/PPy@PDA/TA hydrogel, broad absorption bands were noticed at 3379–3200 cm^−1^, meaning that the phenolic hydroxyl group was well preserved after a chemical reaction. In addition, the characteristic peaks of the functional groups of TA, CS, and the PPy@PDA complex were detected in the FTIR spectrum of the PAM/CS/PPy@PDA/TA hydrogel, indicating the successful synthesis of the PAM/CS/PPy@PDA/TA hydrogel.

Figure 3b,c show the SEM images of the PAM/CS/TA hydrogel and the PAM/CS/PPy@PDA/TA hydrogel, respectively. In contrast, the PAM/CS/PPy@PDA/TA hydrogel had a denser interconnected porous network structure, and no agglomeration of the PPy@PDA complex occurred in the hydrogel network.

### 3.4. Mechanical Properties of the PAM/CS/PPy@PDA/TA Hydrogel

**Figure 4:**
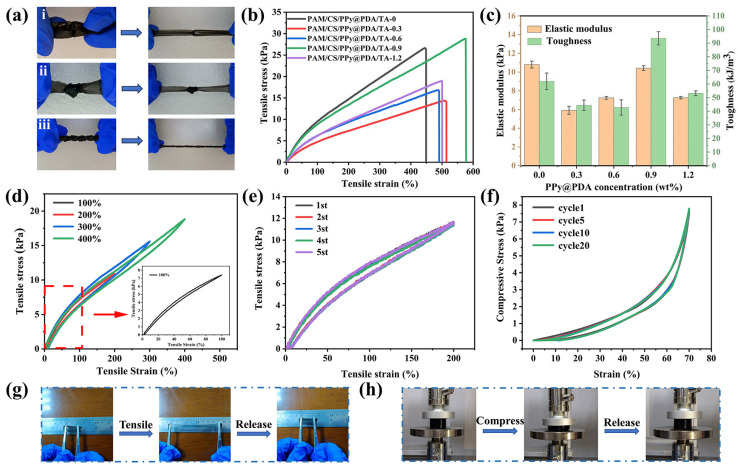
(**a**) Bending (i), knotting (ii), and twisting (iii) of the hydrogel. (**b**) Stress-strain curves of the hydrogels with different PPy@PDA contents. (**c**) Elastic modulus and toughness of the hydrogels with different PPy@PDA contents. (**d**) Cyclic tensile curves of the PAM/CS/PPy@PDA/TA-0.9 wt% hydrogel at different strains. (**e**) Five cyclic tensile curves of the PAM/CS/PPy@PDA/TA-0.9 wt% hydrogel at the 200% strain. (**f**) Cyclic loading-unloading compression curves of the PAM/CS/PPy@PDA/TA-0.9 wt% hydrogel at the 70% strain. (**g**,**h**) Changes in hydrogel shape under stretching and compression, respectively.

Excellent mechanical properties and resistance to fatigue fracture are critical factors to ensure the stability and durability of hydrogel-based flexible electronic devices. A series of experiments was conducted to demonstrate the mechanical properties of the hydrogel (Figure 4). The hydrogel could quickly return to its original state without damage after being subjected to bending, knotting, and twisting (Figure 4a), indicating its superior flexibility. To investigate the effects of the PPy@PDA complex on the mechanical properties of the hydrogels with different amounts of the PPy@PDA complex, their mechanical tensile curves were plotted (Figure 4b). As the content of the PPy@PDA complex was raised from 0 wt% to 0.9 wt%, the elongation at the breakage point increased from 444.617% to 575.082%. The effects of different PPy@PDA contents on the elastic modulus and toughness of the PAM/CS/PPy@PDA/TA hydrogels were calculated based on their tensile stress-strain curves (Figure 4c). When the content of the PPy@PDA complex was 0.9 wt%, the highest elastic modulus, and toughness were achieved (10.144 kPa and 96 kJ/m^3^, respectively). When the content of the PPy@PDA complex was 1.2 wt%, the elastic modulus and toughness of the hydrogel decreased drastically. The PPy@PDA complex had multi-site non-covalent bonds with various molecules, allowing rapid rearrangement of the hydrogel and causing reversible interactions with neighboring molecules when molecular chains ruptured during stretching, thus enhancing the mechanical properties of the hydrogel. However, when the PPy@PDA complex was added excessively, the mechanical properties of the hydrogel deteriorated. It might happen because the excessive amount of the PPy@PDA complex led to excessive cross-linking in the hydrogel, hindering the displacement of molecular chains in the hydrogel network; thus, the hydrogel easily ruptured under an external force. The elastic modulus of the PPy@PDA complex containing hydrogels was significantly reduced compared to those without the PPy@PDA complex, probably because the PPy@PDA complex introduced more catechol groups into the system. It would affect the production of free radicals in APS and thus affect the chemical cross-linking density during the gelation process [59]. When the strain was raised from 100% to 400% during continuous loading-unloading processes, the hysteresis loop of the PAM/CS/PPy@PDA/TA-0.9 wt% hydrogel also gradually increased (Figure 4d). This might be because when the hydrogel was stretched, multiple interactions and dynamic cross-linking between CS and the PPy@PDA complex, hydrogen bonding between TA and Fe^3+^, electrostatic interactions, metal-ligand bonding, and non-covalent bonding dissipated some energy during the breaking and reorganization of the loops. It can be seen from Figure 4e that under tensile cycling at the 200% strain, the dissipated energy of the hydrogel was low and did not change significantly over five cycles, indicating that the hydrogel had excellent self-recovery properties over the human motion range (0–75%).

The compressive properties of the hydrogels were also investigated. Figure 4f shows the strain-stress curves of the PAM/CS/PPy@PDA/TA-0.9 wt% hydrogel under continuous loading-unloading compression tests at a constant strain of 70%. Significant hysteresis loop fluxes were observed in each loading-unloading cycle. The hysteresis loops of the 20 cycles used in this experiment almost overlapped, indicating that the hydrogel had certain fatigue resistance and resilience. In addition, the PAM/CS/PPy@PDA/TA-0.9 wt% hydrogel could recover its initial shape after a certain strain under tension and compression (Figure 4g,h).

### 3.5. Self-Adhesion Properties

**Figure 5:**
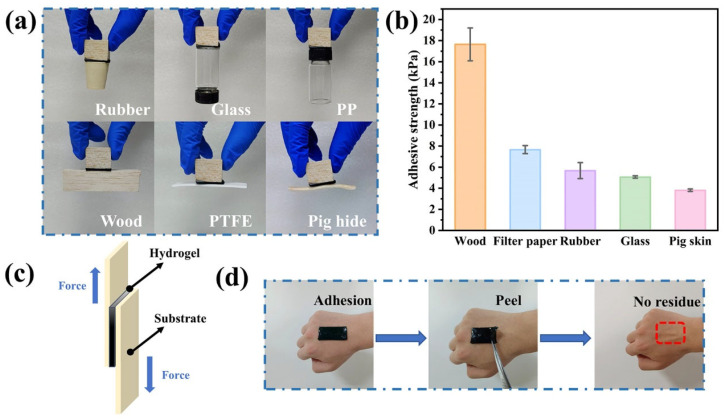
(**a**) Adhesion of the hydrogel to different substrates. (**b**) Adhesion strengths of the hydrogel on different substrate surfaces. (**c**) Lap shear test. (**d**) Removal of the hydrogel from human skin.

The hydrogel exhibited excellent self-adhesive properties on different substrate surfaces, such as glass, wood, plastics, and rubber, by forming hydrogen bonds between abundant catechol groups in TA and hydroxyl, amino, or thiol groups (biological tissues) (Figure 5a). To systematically evaluate the adhesive properties of the hydrogel, its adhesive strengths on different substrate surfaces were measured by lap shear tests (Figure 5c). The hydrogel had a high adhesion strength of 17.646 kPa on the wood surface (Figure 5b), and the corresponding values for filter papers, rubber, glass, and pig skin were 7.664, 5.678, 5.068, and 3.815 kPa, respectively, and this difference in adhesion strengths on different substrates was due to different interfacial interactions. In addition, the hydrogel could adhere to human skin without causing any irritation and could be easily removed without causing pain (Figure 5d). Hence, the as-prepared hydrogel can be applied to smart skin patch sensors.

### 3.6. Strain Sensitivity of the PAM/CS/PPy@PDA/TA Hydrogel

**Figure 6:**
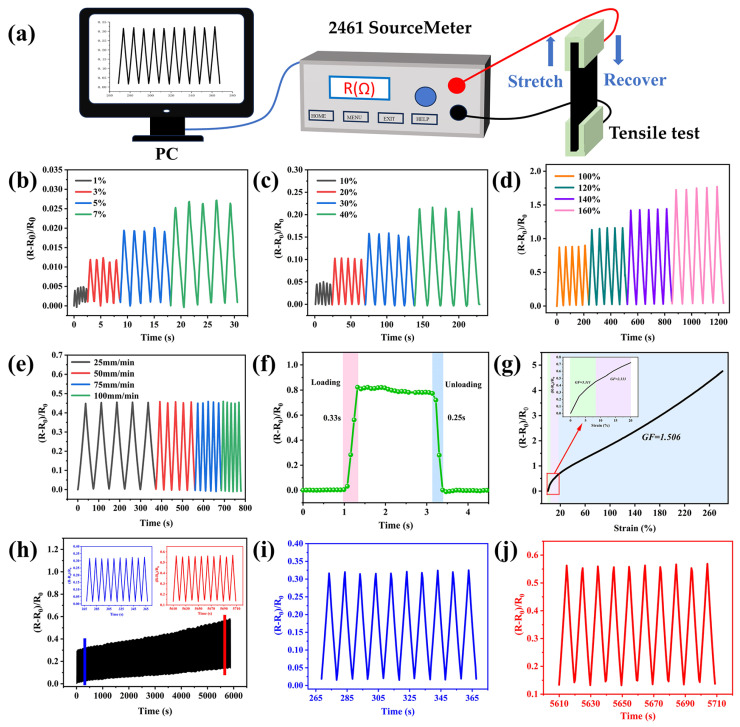
(**a**) Schematic diagram of strain sensitivity test. (**b**–**e**) Relative resistance responses of the hydrogel during five stretching-recovery cycles under small strains, medium strains, large strains, and different rates, respectively. (**f**) Response and recovery times of the hydrogel at 60% strain. (**g**) Relative resistance curve in the 0–280% strain range. (**h**) Relative resistance change curve of the hydrogel after about 550 loading-unloading cycles at 50% strain. (**i**,**j**) Corresponding relative resistance change curves for 10 loading-unloading cycles starting at 265 s and 5610 s in subfigure (**h**), respectively.

As the PAM/CS/PPy@PDA/TA-0.9 wt% hydrogel has good stretchability, excellent adhesion properties, and superb resistive responses, it can be used in strain sensors. Figure 6a is a schematic diagram of the strain sensitivity test. Figure 6b–d shows the changes in the relative resistance of the strain sensor during five stretching-recovery cycles under small, medium, and large strains, respectively. During the stretching-recovery cycles, the hydrogel-based sensor remained stable and exhibited excellent resistance recovery and reproducibility. Moreover, the hydrogel-based sensor responded quickly at different stretching rates and had stable resistance signals (Figure 6e). The PAM/CS/PPy@PDA/TA-0.9 wt% hydrogel-based sensor enabled a fast loading-to-unloading process at 60% strain (Figure 6f). The monitoring process detected almost no signal lag, highlighting the consistency of the hydrogel-based sensor in capturing electric signals under the applied strain. The response time and recovery time of the hydrogel-based sensor were 0.33 s and 0.25 s, respectively. The sensitivities of the GF in strain ranges of 0–8%, 8–20%, and 20–280% were 5.311, 2.333, and 1.506, respectively (Figure 6g). Hence, the sensor had high sensitivity, especially in the small strain range. Durability and stability are crucial factors in using a sensor for a long period. Therefore, the durability and stability of the hydrogel-based sensor were tested by measuring the changes in its relative resistance during cyclic stretching-recovery cycles at 50% strain (Figure 6h). The ΔR/R_0_ value of the hydrogel-based sensor remained stable for the first 550 cycles and then increased slightly (Figure 6i,j) due to the effect of water loss, demonstrating the high repeatability, stability, and durability of the hydrogel-based sensor.

### 3.7. Application of PAM/CS/PPy@PDA/TA Hydrogels in Wearable Electronic Devices

**Figure 7:**
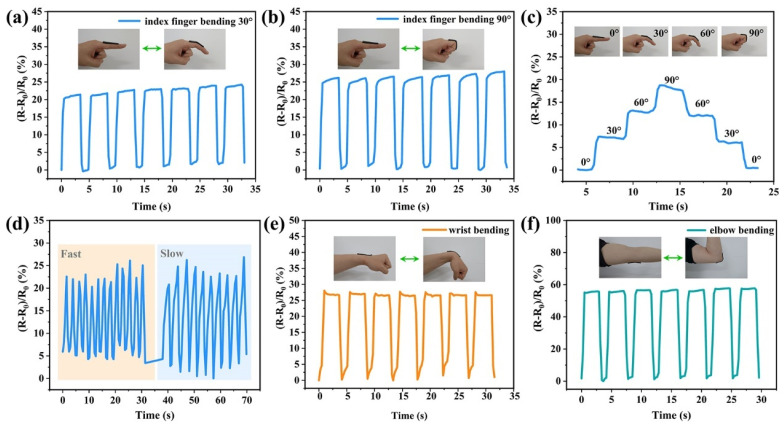
Human motion monitoring by the hydrogel-based sensor: (**a**–**d**) finger movement. (**e**) wrist bending, and (**f**) elbow bending.

The PAM/CS/PPy@PDA/TA-0.9 wt% hydrogel-based strain sensor, due to its excellent mechanical properties, good adhesion properties, high sensitivity, fast response, and superb stability, has great potential in human motion monitoring. The PAM/CS/PPy@PDA/TA-0.9 wt% hydrogel-based strain sensor was used to monitor the movements of all parts of the human body in real time (Figure 7). It can be seen from Figure 7a–c that the relative resistance of the sensor increased as the bending angle of the finger increased; therefore, different bending angles of the finger could be distinguished based on the peak intensities of response signals. It can be seen from Figure 7d that the resistance of the sensor changed steadily when the finger moved slowly or quickly back and forth, and a stable response signal was monitored, demonstrating the high strain sensitivity, fast response, and reliability of the hydrogel-based sensor. The hydrogel-based strain sensor was also used to monitor wrist (Figure 7e) and elbow (Figure 7f) movements. The PAM/CS/PPy@PDA/TA-0.9 wt% hydrogel-based sensor could distinguish the bending behaviors of different joints by comparing the resistance response signals generated from the movements of the finger, the wrist, and the elbow. Due to the excellent self-adhesive properties of the hydrogel, the sensor was successfully attached to human skin, and it transmitted stable electrical signals even during intense body movements and repeated deformation.

Therefore, the PAM/CS/PPy@PDA/TA-0.9 wt% hydrogel-based flexible strain sensor exhibited good response performance and stability for human motion monitoring; hence, it has significant potential in wearable electronic devices.

**Figure 8:**
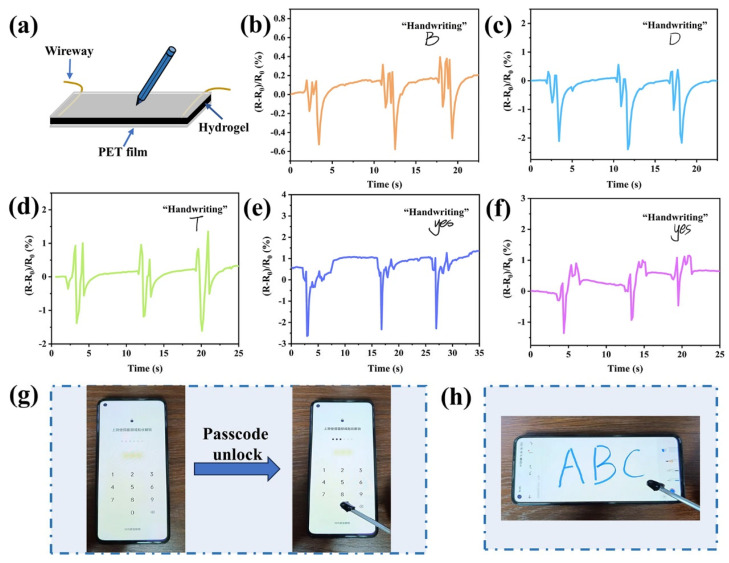
Handwriting recognition by hydrogel-based sensor: (**a**) schematic diagram of the sensor, (**b**–**d**) different letters (B, D, and T) written by the volunteer, (**e**,**f**) the same word “yes” written by different volunteers, and (**g**,**h**) unlocking a cell phone and writing on the phone screen using the hydrogel-based stylus, respectively.

The PAM/CS/PPy@PDA/TA-0.9 wt% hydrogel-based sensor was also used for handwriting recognition (Figure 8a). As each person has a different writing habit, writing strength, and writing speed, unique waveforms are typically generated from their writing. It can be seen from Figure 8b–f that different words written by the volunteers had different waveforms and the same word written by the volunteers also had different waveforms, suggesting that the hydrogel has great potential in anti-counterfeiting applications. In addition, the PAM/CS/PPy@PDA/TA-0.9 wt% hydrogel was transformed into a stylus, and the stylus could control the phone screen, unlock the phone, and write on the phone (Figure 8g,h). Hence, this hydrogel-based sensor can be successfully used in human-computer interaction applications.

## 4. Summary

The ultrafast polymerization of a hydrogel (1–3 min) was achieved at room temperature by constructing a dynamic redox system of TA-Fe^3+^. The mechanical properties of the hydrogel were significantly improved after the incorporation of the PPy@PDA complex. The PAM/CS/PPy@PDA/TA-0.9 wt% hydrogel manifested good adhesion to different materials, such as wood, filter papers, pig skin, and rubber. Interactions between hydrogen bonds and chain entanglements in the hydrogel resulted in reliable mechanical properties (elongation = 575.082% and tensile strength = 28.814 kPa) and excellent strain sensitivity. In addition, the hydrogel had a low detection limit, high sensitivity at small strains (GF = 5.311 at strain = 0–8%), and fast response time (0.33 s) and recovery time (0.25 s). The PAM/CS/PPy@PDA/TA-0.9 wt% hydrogel-based sensor exhibited good response performance and high stability for human motion monitoring. It could also be applied to electronic skins and handwriting recognition. Hence, the current work opens new directions to the development of wearable sensors, smart robots, and electronic skins.

## Figures and Tables

**Table 1:** Composition of elements C, O, and N in PPy and the PPy@PDA complex.

Sample	C (at%)	O (at%)	N (at%)
PPy	81.08	15.83	3.09
PPy@PDA	79.04	19.1	1.86

## Data Availability

Data are contained within the article.
